# Predictive value of brain atrophy, serum biomarkers and information processing speed for early disease progression in multiple sclerosis

**DOI:** 10.3389/fneur.2023.1223220

**Published:** 2023-07-25

**Authors:** Magdalena Oset, Małgorzata Domowicz, Paula Wildner, Małgorzata Siger, Iwona Karlińska, Mariusz Stasiołek, Mariola Świderek-Matysiak

**Affiliations:** Department of Neurology, Medical University of Lodz, Lodz, Poland

**Keywords:** multiple sclerosis, predictive factors, neurofilament light chain, bicaudate ratio, third ventricle width

## Abstract

**Introduction:**

Multiple sclerosis (MS) is a chronic autoimmune-mediated demyelinating disease of the central nervous system (CNS). A clinical presentation of the disease is highly differentiated even from the earliest stages of the disease. The application of stratifying tests in clinical practice would allow for improving clinical decision-making including a proper assessment of treatment benefit/risk balance.

**Methods:**

This prospective study included patients with MS diagnosed up to 1 year before recruitment. We analyzed serum biomarkers such as CXCL13, CHI3L1, OPN, IL-6, and GFAP and neurofilament light chains (NfLs); brain MRI parameters of linear atrophy such as bicaudate ratio (BCR), third ventricle width (TVW); and information processing speed were measured using the Symbol Digit Modalities Test (SDMT) during the 2 years follow-up.

**Results:**

The study included a total of 50 patients recruited shortly after the diagnosis of MS diagnosis (median 0 months; range 0–11 months), and the mean time of observation was 28 months (SD = 4.75). We observed a statistically significant increase in the EDSS score (Wilcoxon test: *Z* = 3.06, *p* = 0.002), BCR (Wilcoxon test: *Z* = 4.66, *p* < 0.001), and TVW (Wilcoxon test: *Z* = 2.84, *p* = 0.005) after 2 years of disease. Patients who had a significantly higher baseline level of NfL suffered from a more severe disease course as per the EDSS score (Mann–Whitney U-test: *U* = 107, *Z* = −2,74, *p* = 0.006) and presence of relapse (Mann–Whitney U-test: *U* = 188, *Z* = −2.01, *p* = 0.044). In the logistic regression model, none of the parameters was a significant predictor for the achieving of no evidence of disease activity status (NEDA). In the model considering all assessed parameters, only the level of NfL had a significant impact on disease progression, measured as the increase in EDSS (logistic regression: β = 0.002, *p* = 0.017).

**Conclusion:**

We confirmed that NfL levels in serum are associated with more active disease. Moreover, we found that TVW at the time of diagnosis was associated with an impairment in cognitive function measured by information processing speed at the end of the 2-year observation. The inclusion of serum NfL and TVW assessment early in the disease may be a good predictor of disease progression independent of NEDA.

## 1. Introduction

Multiple sclerosis (MS) is a chronic autoimmune-mediated demyelinating disease of the central nervous system (CNS). The disease mainly affects young adults and leads to physical disability and cognitive impairment, causing disturbances in social activities, employment, and quality of life. The clinical course of the disease is heterogeneous and varies widely between patients. Therefore, an awareness of factors associated potentially with higher activity and rapid progression of disability are crucial for optimal care planning (1). Taking into consideration the broadening spectrum of highly efficient disease-modifying therapies (DMTs), many clinical, laboratory, and radiological markers are examined for their predictive value in indicating patients at risk of more rapid disease progression.

Patient characteristics, such as age over 35 years old, result of the Expanded Disability Status Score (EDSS) over 3, and first symptom presentation in the form of the pyramidal signs, indicate a higher risk of development of an aggressive disease (2). Moreover, several biomarkers are considered for their utility in predicting progression of the disease. The presence of oligoclonal bands (OCBs) in the cerebrospinal fluid (CSF) is a diagnostic marker (3) and a negative predictor of MS evolution, indicating a greater progression in OCB-positive patients (4). Moreover, a measure of intrathecal production of kappa-free light chains is gaining interest as a quantitative alternative to OCB which is also a valuable diagnostic tool (5, 6) and predicts early activity of the disease (7). Currently, one of the most promising biomarkers is neurofilaments, which are cytoskeletal proteins released from damaged axons into the CSF and the blood. The neurofilament light chain (NfL) level is correlated with the CNS inflammation in MS, with higher levels during acute relapse, but it is also helpful in assessing the neurodegenerative process, indicating the progression from relapsing–remitting MS (RRMS) to secondary progressive MS (SPMS). A decrease in NfL levels is postulated to be one of the markers of DMT effectiveness (8, 9). Glial fibrillary acidic protein (GFAP), considered as a marker of astrocyte damage, was found in MS plaques, and higher levels of GFAP are associated with greater disease disability and frequency of relapses (10, 11). GFAP was found to be significantly higher in patients with primary progressive MS (PPMS) even after adjusting for age; however, there are conflicting results about its levels during relapses. Additionally, it was reported that levels of serum GFAP were positively associated with the white matter lesion load and negatively correlated with white matter (WM), gray matter (GM), and cortical GM volumes (11). Moreover, a significant correlation was observed between serum GFAP levels and the intensity of gadolinium-enhancement (Gd) lesions in brain magnetic resonance imaging (MRI) (12). The next potential biomarker is C-X-C motif chemokine ligand 13 (CXCL13). Although increased levels of CXCL13 were described in other neurological conditions including infections, its increased CSF levels have been correlated with worse prognosis and exacerbations in RRMS and conversion from clinically isolated syndrome (CIS) to clinically define MS (13). Moreover, the serum CXCL13 level was proposed as a treatment response marker to teriflunomide as it was significantly lower in patients achieving no evidence of disease activity (NEDA-3) status after 1 year of therapy (14). Higher serum IL-6 concentrations have been found in RRMS patients associated with the age of onset of the disease (15). Another promising but non-specific biomarker is chitinase-3-like-1 precursor (CHI3L1); CHI3L1 serum and CSF levels were found to be increased as the disease progressed. CHI3L1 is also associated with conversion from CIS to RRMS and is correlated with a higher relapse rate (16). Although some studies reported that the levels of osteopontin (OPN) were higher in the blood and CSF of MS patients and correlated with relapse rate and disease severity (17, 18), others did not confirm these findings. Therefore, it is supposed that OPN correlates with disease susceptibility rather than severity (1).

Brain MRI is crucial in the diagnostic process and for the evaluation of disease activity and response to treatment in MS. The conventional assessment of MRI includes WM lesion load, presence of new or enlarged T2 lesions, and Gd-enhancing lesion count. However, it is of considerable interest to find MRI markers of disease progression. Another MRI measure is the T1 lesion load, which reflects old and inactive lesions of a severely damaged tissue. On the other side, several advanced MRI prognostic markers were proposed (14). One of those is global brain atrophy, which is associated with long-term disability. Advanced MRI procedures allow also for the meticulous investigation of specific brain regions such as the cortical gray matter (concerning mostly cortical lesions and cortical atrophy) and deep gray matter including thalamic volume, which is also considered as an independent predictor of disease progression (14). Nevertheless, the non-standard MRI techniques are hard to perform in everyday practice as most of them require sophisticated post-processing of MRI images. To meet the need for simple but reliable measures of brain atrophy, linear atrophy parameters such as the bicaudate ratio (BCR) and the third ventricle width (TVW) were proposed (15). BCR is a surrogate marker of global supra-tentorial white matter atrophy (16), and the TVW corresponds with thalamic atrophy (17). Recently, it was shown that these two-dimensional parameters, which are easy and quick to measure in everyday practice, may be useful in the prognosis of disease progression (18). Recently, the cutoff scores for these parameters reflecting the degree of brain volume loss were suggested to be helpful in classifying the patients in association with physical disability and information processing speed (19).

Moreover, a decrease in cognitive functions, especially in information processing speed measured by the result of the Symbol Digit Modalities Test (SDMT), was shown to be a significant and independent predictor of physical disability progression (20). Additionally, SDMT results were reported as a predictor of employment status and therefore associated with the quality of life of patients with MS (21, 22). Several other measures of disease activity were proposed as therapeutic goals, one of which is no evidence of disease activity-3 (NEDA-3) defined as no relapse, no disability worsening, and no MRI activity (defined as no new or enlarging T2-weighted lesions or Gd-enhancing lesions on brain MRI) during the time of observation.

Finding available and easy tests in routine clinical practice, preferably based on peripheral blood examination, to evaluate risk of the progression at the beginning of the disease would allow for earlier intensification and optimized treatment. The aim of this study was the prospective multiparametric assessment of patients newly diagnosed with MS (up to 12 months after diagnosis) to identify factors that were prognostic for disease activity and progression during a 2-year follow-up (23).

## 2. Materials and methods

### 2.1. Clinical and radiological assessment of the study population

This was a prospective study including RRMS patients who underwent a routine diagnostic MS process at the Department of Neurology Medical University of Lodz, Poland between 2018 and 2022. Adult (above 18 years of age) patients with RRMS diagnosis based on the 2017 revised McDonald criteria established up to 12 months before the recruitment were eligible for the study. After obtaining an informed consent from the participants for the study, participants were followed prospectively for 24 months. Data about clinical relapses, follow-up MRI results, and DMTs exposure were collected from the hospital database. The patient neurological status was examined and scored using the Expanded Disability Status Score (EDSS). Moreover, during the follow-up examination (after 2 years of observation), the patients completed a questionnaire prepared by the authors regarding their perceived health status, quality of life, and severity of symptoms. At the follow-up visit, every patient was assessed for obtaining NEDA-3 status defined as no relapse, no disability worsening measured by an increase in the EDSS score, and no MRI activity (no new or enlarged T2 lesions and no T1 Gd-enhancing lesions) during the 2 years of the study.

Cognitive functions were evaluated using the Symbol Digit Modalities Test (SDMT). The patients were assessed using a written version of SDMT at the baseline visit and after 2 years of follow-up visits. Several patients completed the SDMT test as a part of other evaluations between the assessments; nevertheless, the minimum period between repetition of the test was 3 months. We identified cognitive dysfunction when patients scored less than the 5th percentile value of the healthy control group according to Polish validation of BICAMS (< 40 points) (24).

The patients underwent a routine MRI of the brain performed on the 3T scanner (Vida, Siemens, Munich, Germany) at the beginning of the study and once for every year of observation. The MRI protocol included the following sequences: a high-resolution axial 3-dimensional (3D) T1-weighted magnetization-prepared rapid gradient-echo (MPRAGE) (repetition time (TR) = 2,200 ms, echo time (TE) =2.46 ms, inversion time (TI) = 900 ms, field of view (FOV) = 256, number of slices = 167, and pixel size = 1 × 1 × 1 mm), sagittal isotropic 3D T2-weighted fluid-attenuated inversion recovery (FLAIR) (TR = 2,560 ms, TE = 135 ms, TI = 6,700 ms, FOV=256, and number of slices = 192), proton density PD/T_2_-weighted (TR = 2,560 ms, TE1/TE2 = 90/30 ms, FOV=256, number of slices = 46, and slice thickness = 3.0 mm), and 3D T_1_-MPARAGE (repetition time (TR) = 2,200 ms, echo time (TE) =2.46 ms, inversion time (TI) = 900 ms, field of view (FOV) = 256, number of slices = 167, and pixel size = 1 × 1 × 1 mm) after intravenous contrast administration (gadolinium-based, gadobutrol 0.1 mmol/kg body weight).

**Figure 1:**
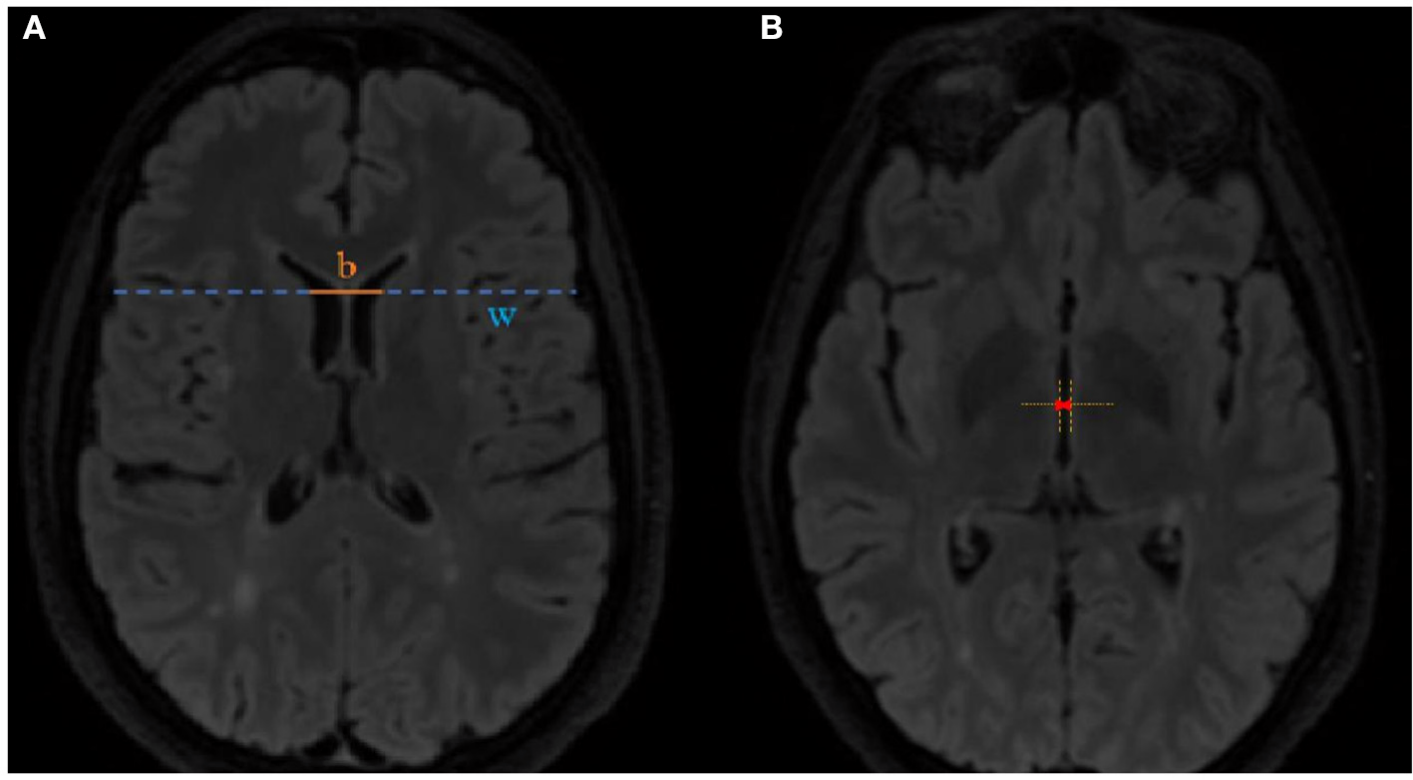
Method of **(A)** bicaudate ratio BC*R* = $\frac{b}{w}$ and **(B)** third ventricle width measurement.

MRI scans were assessed for new or enlarged T2 lesions and T1 contrast enhancement, and linear parameters of brain atrophy such as the BCR and the TVW were also calculated. The BCR was calculated at the axial FLAIR image at the level of caudate nuclei by dividing the inter caudate distance by the whole-brain width at the same level (shown in Figure 1A). The TVW was measured at the axial FLAIR image at the level where it is the widest (Figure 1B).

The protocol of the study was reviewed and approved by the Local Ethics Committee of the Medical University of Lodz (RNN/376/18/KE). The patients provided their written informed consent to participate in this study.

### 2.2. CXCL13, GFAP, CHI3L1, OPN, IL-6, and NF-light assessment in serum

Concentrations of human CXCL13, CHI3L1, OPN, and IL-6 [Biorbyt Ltd. (Cambridge, UK)], GFAP [Wuhan EIAab Science (Wuhan, China)], and NfL [Uman Diagnostics (Umea, Sweden)] were measured in serum using appropriate ELISA kits. All reagents were used according to the manufacturer's recommendations. Sera were collected from all patients at the beginning of the study.

In brief, for CXCL13, CHI3L1, OPN, IL-6, GFAP, and NfL measurements, serum samples were applied on plates coated with specific antibodies against a tested antigen, respectively, and the molecular agent present in a sample was bound to the wells. After washing, biotinylated antihuman antibody against tested factor, HRP-conjugated streptavidin and TMB substrate solution were added, and the intensity of the developed color was measured at 450 nm on the EPOCH (BioTek) microplate reader. The intra-assay validation was performed, and four quality control (QC) samples were included on each plate. All assays were run in duplicates, and the average % coefficient of variation (CV) was 15% or less.

### 2.3. Statistical analysis

Statistical analysis was performed with Statistica 13, StatSoft. Non-parametrical tests were implemented due to the violation of both the normality of data distribution and the heterogeneity of variance assumptions revealed by the Shapiro–Wilk test and Levene's test, respectively. The Wilcoxon test was used to assess the differences in the subsequent analyses of SDMT and linear atrophy parameters. Correlations between parameters were evaluated using the Spearman's R test. Comparisons between groups (patients with an increase in the EDSS score, presence of relapse, subjective deterioration in health reported in the questionnaire, and achieving of NEDA-3 status vs. groups without these features) were performed using the Mann–Whitney U-test. Bonferroni correction was used for *p*-value adjustment for multiple comparisons. Comparisons between NEDA/EDA groups according to the presence of T2 and gadolinium-enhancing lesions and other nominal variables were assessed using the chi-square test or Fisher's exact test, whichever was appropriate. Logistic regression was used to point out prognostic factors for measures of disease activity such as an increase in the EDSS score and achieving NEDA-3 status. A ROC analysis determined the diagnostic values and the optimal cutoff value for the risk of increase in the EDSS score, and cognitive impairment was identified using the Youden index.

## 3. Results

### 3.1. Demographic characteristics

The study included a total of 50 patients, 34 (68%) women and 16 (32%) men with a mean age of 33 years (SD = 8.6). Patients were included in the study shortly after diagnosis (median 0 months and range 0–11 months). The mean time of observation was 28 months (SD = 4.75). All of the patients were oligoclonal band-positive. A total of 30 patients (60%) had already received DMTs on average 4.2 (SD = 2.8) before the inclusion in the study (glatiramer acetate−5 patients, interferon beta−3 patients, teriflunomide−3 patients, and dimethyl fumarate−19 patients). During the last assessment, all the patients have been receiving DMTs (glatiramer acetate−5 patients, dimethyl fumarate−36, fingolimod−1, ocrelizumab−2, teriflunomide−3, natalizumab−1, interferon beta−2 patients. A total of 5 patients (10%) changed DMTs treatment during the study. Among patients receiving DMT, 14 (47%) had Gd-enhancing lesions; on the other hand, only 3 patients (15%) had not received DMT (chi^2^ test: χ = 5.36, *p* = 0.021). The demographic data are presented in Table 1.

**Table 1:** Demographic characteristics of the patients.

	**Mean**	**SD**
Number of female/male	34/16	
Age	32.8	8.39
Time since diagnosis (months)	0 (median)	0–11 (range)
Time of formal education (years)	15.5 (median)	10–32 (range)
Time of observation (months)	28.14	4.75

### 3.2. Serum biomarkers

**Figure 2:**
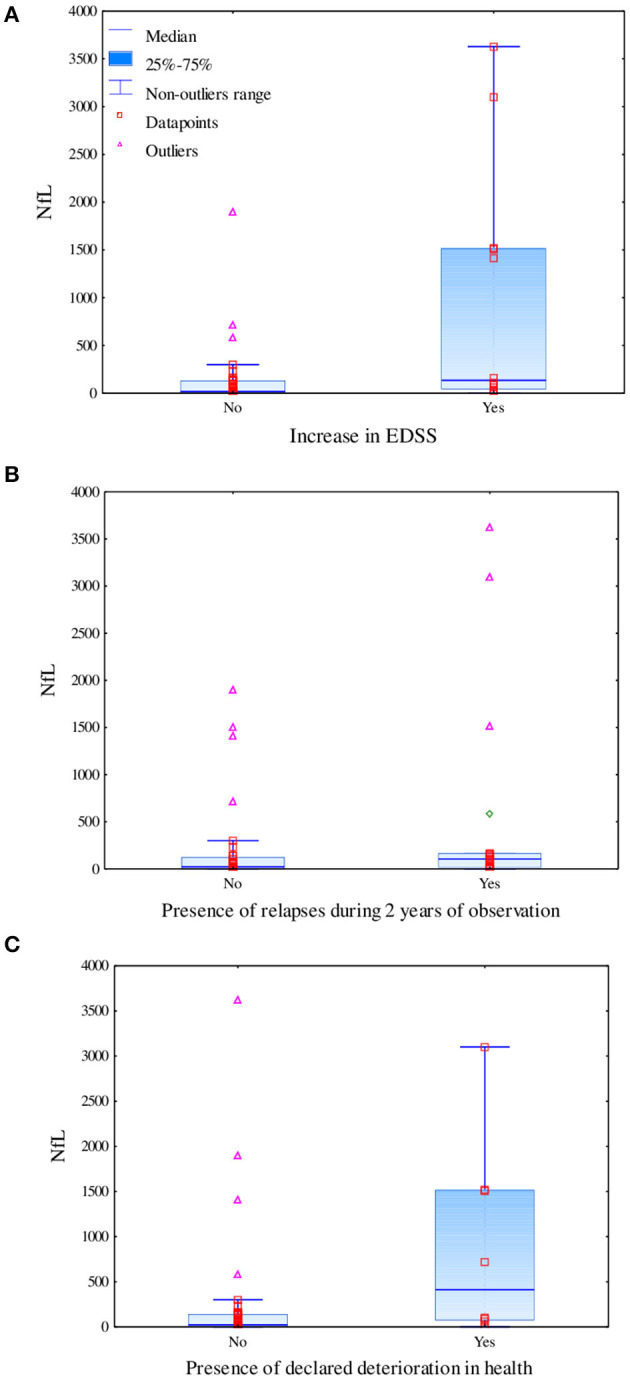
Difference in levels of serum neurofilament light chains based on the increase in the EDSS score measured using the Mann–Whitney test (*U* = 107, *Z* = −2.74, *p* = 0.006) **(A)**, the presence of relapses during 2 years of observation measured using the Mann–Whitney test (*U* = 188, *Z* = −2.01, *p* = 0.044) **(B)**, and the declared deterioration in health measured using the Mann–Whitney test (*U* = 83, *Z* = −2.24, *p* = 0.025) **(C)**.

In the linear regression model, levels of biomarkers were independent of age; however, we found an impact of DMT on levels of CHI3L1 (β = -1,547.418; *p* = 0.062) and IL-6 (β = 41.268; *p* = 0.030). Taking into consideration the results of serum biomarkers, we observed a tendency toward a positive correlation of IL-6 level (*R* = 0.3496, *p* = 0.0128) and negative correlation of OPN level (*R* = −0.2998, *p* = 0.0344), with the result of the SDMT at the baseline assessment observed. Additionally, we observed a tendency toward a positive correlation between the level of IL-6 and BCR at the baseline MRI assessment (*R* = 0.2788, *p* = 0.045). However, after Bonferroni correction for multiple analyses, none of the biomarkers correlated significantly with SDMT or BCR and TVW (results are shown in Table 2). Moreover, patients who had significantly higher levels of NfL at the baseline were characterized by a more severe disease course based on an increase in the EDSS score (Mann–Whitney test: *U* = 107, *Z* = −2.74, *p* = 0.006), presence of relapse (*U* = 188, *Z* = −2.01, *p* = 0.044), and patients own opinion of deterioration in health reported in the questionnaire (*U* = 83, *Z* = −2.24, *p* = 0.025), as shown in Figures 2A–C, respectively.

**Table 2:** Results of Spearman's R tests between the baseline level of serum biomarkers and SDMT or linear atrophy parameters during the study.

	**Baseline assessment**					
	**SDMT**		**BCR**		**TVW**	
	**R**	**p**	**R**	**p**	**R**	**p**
IL-6	0.35	0.013	0.28	0.045	0.11	0.452
OPN	−0.30	0.034	−0.03	0.848	0.15	0.297
CXCL13	−0.11	0.427	0.01	0.929	0.22	0.127
GFAP	−0.07	0.616	−0.07	0.633	−0.02	0.914
NfL	−0.12	0.411	0.10	0.494	0.17	0.252
CHI3L1	−0.10	0.573	−0.01	0.970	0.24	0.097
	**Follow–up assessment**					
	**SDMT**		**BCR**		**TVW**	
	**R**	**p**	**R**	**p**	**R**	**p**
IL-6	0.23	0.109	0.21	0.149	0.11	0.452
OPN	0.01	0.966	−0.02	0.918	0.02	0.890
CXCL13	−0.001	0.995	−0.07	0.632	0.09	0.515
GFAP	0.06	0.671	−0.10	0.491	−0.09	0.542
NfL	0.03	0.826	−0.08	0.583	0.13	0.351
CHI3L1	0.08	0.573	0.06	0.693	0.17	0.233

Statistical significance was defined using a p-level of 0.05, which, after Bonferroni correction with a factor of 6 for multiple comparisons, was equivalent to 0.008.

IL-6, interleukin 6; OPN, osteopontin; CXCL13, C-X-C motif chemokine ligand 13; GFAP, glial fibrillary acidic protein; NfL, neurofilament light chains; SDMT, Symbol Digit Modalities test; BCR, bicaudate ratio.

### 3.3. Linear parameters of brain atrophy and information processing speed analysis

In our cohort, linear parameters of brain atrophy showed progression during the time of the study observation. We observed a statistically significant increase in BCR (Wilcoxon test: *Z* = 4.66, *p* < 0.001) and TVW (*Z* = 2.84, *p* = 0.005). BCR was positively correlated with age at the follow-up (*R* = 0.40, *p* = 0.004), and TVW was positively correlated with age in both assessments (*R* = 0.44, *p* = 0.001 for the baseline and *R* = 0.47, *p* = 0.001 for the follow-up assessment). We performed the Mann–Whitney U-test to check for associations between the presence of baseline Gd-enhancing lesions and the level of serum biomarkers, but after correction for multiple comparisons, none is statistically significant (CXCL13: *p* = 0.513; CHI3L1: *p* = 0.041; GFAP: *p* = 0.712; OPN: *p* = 0.874; IL-6: *p* = 0.073; NfL: *p* = 0.5). SDMT is a reliable measure of information processing speed and may be used solely to assess this modality. As a result of the COVID-19 pandemic and fatigability of the patients, we decided to use only the SDMT to reduce the time burden and simplify the evaluation (25). The result of the SDMT positively correlated with years of education at the follow-up assessment (*R* = 0.37, *p* = 0.008) but not with the age of the patients (*R* =−0.19, *p* = 0.179). Interestingly, a slight but statistically significant increase in the result of SDMT (Z = 2.07, *p* = 0.038) at the follow-up was reported (data presented in Table 3). The higher result of the SDMT was associated with receiving DMTs early, before the inclusion in the study (*U* = 54, Z = 2.27, *p* = 0.023). At the baseline assessment, eight patients (16%) and, after 2 years, seven patients (14%) underperformed in the SDMT (scored < 40 points).

**Table 3:** Results of linear atrophy parameters, EDSS and SDMT, in both assessments and the level of statistical significance in the difference between the assessments measured using the Wilcoxon test.

	**Baseline assessment**			
	**BCR**	**TVW**	**EDSS**	**SDMT**
Median	0.125	3.950	1	50.5
	**Follow-up assessment**			
	**BCR**	**TVW**	**EDSS**	**SDMT**
Median	0.138	4.000	1	52.5
p (Wilcoxon test)	***p*** **<** **0.001**	***p*** **=** **0.005**	***p*** **=** **0.002**	*p* = 0.038

Statistical significance was defined using an α level of 0.05, which, after Bonferroni correction with a factor of 4 for multiple comparisons, was equivalent to 0.013.

BCR, bicaudate ratio; TVW, third ventricle width; EDSS, Expanded Disability Status Scale; SDMT, Symbol Digit Modalities Test.

The values in bold are statistically significant.

**Figure 3:**
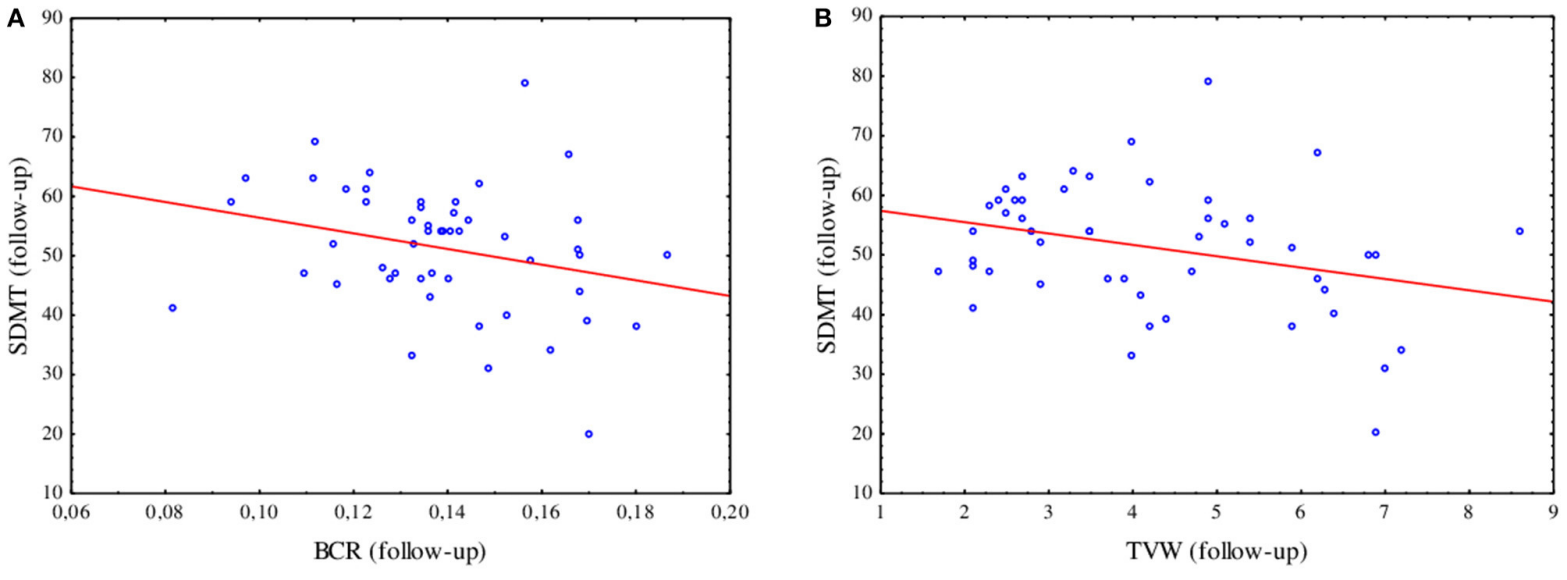
Spearman's R correlation between the SDMT and BCR (*R* = −0.32, *p* = 0.025) **(A)** and TVW (*R* = −0.28, *p* = 0.049) **(B)** at the follow-up assessment.

At the follow-up, a negative correlation was found between the results of SDMT and BCR (*R* = −0.32, *p* = 0.025) as well as between SDMT and TVW (*R* = −0.28, *p* = 0.049), as shown in Figure 3. In the logistic regression model including linear parameters of atrophy and serum biomarkers, only TVW remained as a predictor of impairment in information processing speed (β = 0.720 *p* = 0.030). The ROC analysis revealed an optimal TVW cutoff value of 5.2 mm (Youden index = 0.55) for discriminating the impairment in information processing speed after 2 years of observation, which resulted in 71.4% sensitivity and 83.7% specificity with an area under the curve (AUC) of 0.764 (*p* = 0.004).

### 3.4. Assessment of disease activity and progression

At the follow-up, we found an increase in the EDSS score (*Z* = 3.06, *p* = 0.002). In our cohort, NEDA-3 after 2 years of disease was achieved by 16 patients (32%)−11 women and 5 men. The group with the evidence of disease activity (EDA) consisted of 23 women and 11 men, and the difference between groups was not significant (*p* = 0.602). We did not find any difference between the NEDA and the EDA groups in the baseline EDSS (*p* = 0.148) although the mean EDSS score in the NEDA group was 0.90 and in the EDA group was 1.25. We also did not find statistically significant differences in the number of T2 lesions (*p* = 0.138) and the presence of Gd-enhancing lesions (*p* = 0.277) in the baseline MRI of the brain in the NEDA and EDA groups. Patients who achieved the NEDA-3 status were older (*U* = 133, *Z* = 2.88, *p* = 0.004) and had significantly higher BCR (*U* = 157, *Z* = 2.38, *p* = 0.017) and TVW (*U* = 168, *Z* = 2.14, *p* = 0.032) in the follow-up assessed MRI scans as compared to EDA patients. However, no significant differences in BCR and TVW results were found at the baseline MRI assessment between patient with NEDA and EDA (respectively, *p* = 0.116; *p* = 0.131). In the logistic regression model, none of the parameters was a significant predictor for the achieving of NEDA-3 status. Only the level of NfL had a significant impact on disease progression measured by the increase in EDSS (β = 0.002, *p* = 0.017). The ROC analysis determined the cutoff value for the NfL level of 24.27 pg/ml (Youden index = 0.47), which allowed for 91.7% sensitivity and 55.3% specificity in considering the risk of increase in the EDSS score (AUC = 0.765, *p* < 0.001).

## 4. Discussion

In this study, we tried to identify predictors of disease progression at the early stage of relapsing–remitting MS. During our follow-up of 2 years from diagnosis, we reported a slow accumulation of disability and increasing atrophy of the brain. Nowadays, one of the most established predictors of future disability is the presence of OCB in CSF. In our cohort, all patients were OCB positive; however, we were not able to assess the levels of KFLC, which is an important limitation, as they provide an additional value to the diagnostic performance and prediction of early accumulation of disability and higher disease activity.

We included several serum biomarkers which bear a potential value in the prognosis of disease severity. Although after correction for multiple analyses, none of the biomarkers correlated significantly with SDMT or BCR and TVW, and we observed a tendency toward association of proinflammatory IL-6 with more severe atrophy of the brain and higher level of OPN with a decrease in information processing speed measured by the SDMT. For IL-6, similar results were noted in cohorts of patients with ischemic risk and Alzheimer's disease. Higher levels of IL-6 were associated with lower total brain volume than expected for corresponding age group in the cohort from the Framingham heart study (26). Moreover, higher OPN levels were reported in dementia, namely Alzheimer's disease and vascular dementia (27). Additionally, we confirmed the results from previous studies that NfL levels in serum are associated with more active disease (28, 29) as we noted higher levels of this biomarker in patients with relapses during the time of the study and also with higher EDSS at the end of the observation. Currently, the most sensitive tool to evaluate the level of NfL in serum and blood is single-molecule array (SIMOA), which was validated in multiple sclerosis and shows a good correlation between NfL levels in blood and CSF. The high cost and limited access to NfL assessment using SIMOA limit the wider use of NfL assessment, and for this reason, we decided to use ELISA, whose results correlate with those of SIMOA (30), as the most accessible method. In the regression model including all assessed parameters, in our study, only NfL remained as a significant predictor of an increase in the EDSS score after 2 years of disease with the cutoff value of 24.27 pg/ml for the NfL level, which allowed for 91.7% sensitivity and 55.3% specificity in considering the risk of an increase in the EDSS score. A wide range of NfL concentrations both in controls and in MS patients cohorts are observed, and additional prospective, large-scale, multi-centers using the same assay studies are needed (31).

In our study, we observed deviating results concerning information processing speed measured by the result of the SDMT. According to previous observations, this test is highly sensitive but non-specific in MS (32). Nevertheless, it seems to be the most effective single tool to assess cognition even in the initial stages of the disease (33). Despite the result of previous studies which reported that SDMT has a minimal practice effect even if administered at monthly intervals (34), we observed a significant increase in the result of this test, which corresponds to reports from more recent studies (35). Regardless of the practice effect shown by an increased result of the SDMT, we still confirmed that this test may be useful in predicting disease progression as the linear parameters of atrophy correlated negatively with the result of SDMT. Several previous studies confirmed that TWV, as a measure of thalamic atrophy, is a good predictor of cognitive impairment (17, 36, 37). Nishizawa et el. reported that a cutoff value of 5.56 mm for TVW results in 86% sensitivity and 77% specificity in discriminating patients with severe brain volume loss associated with higher EDSS and lower processing speed scores (19). We used this cutoff point in our group, which resulted in a sensitivity of 42.86% and specificity of 88.37%. In our cohort study, the optimal cutoff value of 5.2 mm resulted in 71.4% sensitivity and 83.7% specificity in discriminating the impairment of information processing speed measured by SDMT after 2 years of observation. However, in our study, linear parameters of brain atrophy such as BCR and TVW did not contribute to predicting accumulation of disability measured by an increase in the EDSS score after 2 years of disease.

Although it was previously reported that a decrease in information processing speed may be a predictor of a higher EDSS score (20), we did not note any correlation between those parameters. In our cohort, patients achieving NEDA-3 status were older and with higher atrophy measures. However, we did not find any association with SDMT or assessed serum biomarkers. It was shown before that achieving NEDA-3 was associated with a slower accumulation of physical disability but without an impact on cognition (38). It was also previously shown that NEDA-3 may not be sufficient as a therapeutic goal in MS as 2-year NEDA-3 does not predict disability after 10 years (39). Furthermore, 58.3% of RRMS patients with 2-year NEDA-3 showed impairment in cognitive function (40). Additionally, the usefulness of NEDA as a therapeutic goal has been questioned as it bears several limitations such as the frequency of evaluation, the quality of MRI scans, and the EDSS assessment (41) and also neglects underlying pathophysiology of the disease resulting in insufficient evidence of its prognostic value in the progression of MS (42).

The limitation of the study includes using only the SDMT in assessing cognition as well as the fact that the test was repeated. Additionally, the levels of serum biomarkers were evaluated only at the beginning of the study.

In conclusion, we suggest that NfL is the most promising biomarker associated with the severity of the disease and accumulation of physical disability at an early stage of MS; however, biomarkers such as IL-6 or OPN need further research. Atrophy of the brain was correlated with decreased information processing speed despite the external factors influencing the result of the SDMT. We assume that the best linear parameter for predicting the risk of cognitive impairment early in the disease course is TVW, as higher results from the initial MRI scan were able to determine impairment in information processing speed. According to the current knowledge, no independent biomarker stands solely in the prediction of the accumulation of disability in MS, and combinations of several biomarkers may be the most appropriate way to evaluate the future course of the disease.

## Data availability statement

The raw data supporting the conclusions of this article will be made available by the authors, without undue reservation.

## Ethics statement

The studies involving human participants were reviewed and approved by local Ethics Committee of Medical University of Lodz (RNN/376/18/KE). The patients/participants provided their written informed consent to participate in this study.

## Author contributions

MŚ-M and MO: conceptualization and design of the study and writing—original draft preparation. MŚ-M, MO, and MSi: methodology. MŚ-M, MO, MD, PW, IK, and MSi: investigation and formal analysis. MO: statistical analysis. MŚ-M, MO, and MSt: writing—reviewing and editing. All authors have read and agreed to the published version of the manuscript.

## Supplementary material

Supplementary Table 1 File of raw data presented results of serum biomarkers, SDMT and linear brain atrophy parameters.
